# Supplementary material for: The Systems Biology Research Tool: evolvable open-source software
Source: BMC Syst Biol. 2008 Jun 29;2:55. doi: 10.1186/1752-0509-2-55 (PMC2446383; doi:10.1186/1752-0509-2-55)
Supplement: Additional file 1 — SBRT Archive. An archive of the current version of the Systems Biology Research Tool. [file 1752-0509-2-55-S1.zip › sbrt-1.4.0/doc/developers_guide/api/sbrt/kernel/math/ConstraintViolationException.html]

ConstraintViolationException


|  |  |  |
| --- | --- | --- |
| **Overview** \| **Package** \| **Class** \| **Use** \| **Tree** \| **Deprecated** \| **Index** \| **Help** | |  |
| **PREV CLASS**   **NEXT CLASS** | **FRAMES**    **NO FRAMES**     **All Classes** |  |
| SUMMARY: NESTED \| FIELD \| CONSTR \| METHOD | DETAIL: FIELD \| CONSTR \| METHOD |  |


---


## sbrt.kernel.math Class ConstraintViolationException

```
java.lang.Object
  java.lang.Throwable
      java.lang.Exception
          java.lang.RuntimeException
              sbrt.kernel.math.ConstraintViolationException
```

**All Implemented Interfaces:**: java.io.Serializable

---

``` public class ConstraintViolationException extends java.lang.RuntimeException ```

This class is used to indicate a constraint violation.

**Author:**
:   This class was written and documented by Jeremiah Wright
    while in the Wagner lab.

**See Also:**: Serialized Form

---

**Constructor Summary**

`ConstraintViolationException(java.lang.Object variable,
Interval bounds,
double violatingValue)`
  
          Constructs a new constraint violation exception.

`ConstraintViolationException(java.lang.Object variable,
Interval bounds,
java.lang.Number violatingValue)`
  
          Constructs a new constraint violation exception.


| **Method Summary** | |
| --- | --- |
| `Interval` | `getBounds()`             Returns the constraints that have been violated. |
| `java.lang.Number` | `getValue()`             Returns the value that has violated its constraints. |
| `java.lang.Object` | `getVariable()`             Returns the variable that has violated its constraints. |

**Methods inherited from class java.lang.Throwable**

`fillInStackTrace, getCause, getLocalizedMessage, getMessage, getStackTrace, initCause, printStackTrace, printStackTrace, printStackTrace, setStackTrace, toString`

**Methods inherited from class java.lang.Object**

`clone, equals, finalize, getClass, hashCode, notify, notifyAll, wait, wait, wait`

**Constructor Detail**

### ConstraintViolationException

```
public ConstraintViolationException(java.lang.Object variable,
                                    Interval bounds,
                                    double violatingValue)
```

:   Constructs a new constraint violation exception.

    **Parameters:**: `variable` - the variable whose constraints have been violated.: `bounds` - the constraints that have been violated.: `violatingValue` - the value that violates its constraints.

---


### ConstraintViolationException

```
public ConstraintViolationException(java.lang.Object variable,
                                    Interval bounds,
                                    java.lang.Number violatingValue)
```

:   Constructs a new constraint violation exception.

    **Parameters:**: `variable` - the variable whose constraints have been violated.: `bounds` - the constraints that have been violated.: `violatingValue` - the value that violates its constraints.


**Method Detail**

### getVariable

```
public java.lang.Object getVariable()
```

:   Returns the variable that has violated its constraints.

    :   **Returns:**: the variable that has violated its constraints.

---


### getBounds

```
public Interval getBounds()
```

:   Returns the constraints that have been violated.

    :   **Returns:**: the constraints that have been violated.

---


### getValue

```
public java.lang.Number getValue()
```

:   Returns the value that has violated its constraints.

    :   **Returns:**: the value that has violated its constraints.


---


|  |  |  |
| --- | --- | --- |
| **Overview** \| **Package** \| **Class** \| **Use** \| **Tree** \| **Deprecated** \| **Index** \| **Help** | |  |
| **PREV CLASS**   **NEXT CLASS** | **FRAMES**    **NO FRAMES**     **All Classes** |  |
| SUMMARY: NESTED \| FIELD \| CONSTR \| METHOD | DETAIL: FIELD \| CONSTR \| METHOD |  |


---
